# Overexpression of GREM1 Improves the Survival Capacity of Aged Cardiac Mesenchymal Progenitor Cells via Upregulation of the ERK/NRF2-Associated Antioxidant Signal Pathway

**DOI:** 10.3390/cells12081203

**Published:** 2023-04-21

**Authors:** Gurleen Kaur, Xiaoliang Wang, Xiuchun Li, Hannah Ong, Xiangfei He, Chuanxi Cai

**Affiliations:** 1 Department of Molecular and Cellular Physiology, Department of Medicine, Albany Medical College, Albany, NY 12208, USA; gkaur9@bwh.harvard.edu (G.K.); fmw4te@virginia.edu (X.W.); lxchosu@hotmail.com (X.L.); 2 Department of Medicine, Brigham and Women’s Hospital, Harvard Medical School, Boston, MA 02115, USA; 3 Division of Surgical Sciences, Department of Surgery, University of Virginia, Charlottesville, VA 22903, USA; 4 Department of Surgery, Davis Heart and Lung Research Institute, The Ohio State University Wexner Medical Center, Columbus, OH 43210, USA; hannahong1234@gmail.com (H.O.); xiangfeihe2017@hotmail.com (X.H.)

**Keywords:** GREM1, human cardiac mesenchymal progenitor cells (hMPCs), senescence, oxidative stress, antioxidant, cell survival

## Abstract

Ischemic heart disease is the leading cause of mortality in the United States. Progenitor cell therapy can restore myocardial structure and function. However, its efficacy is severely limited by cell aging and senescence. Gremlin-1 (GREM1), a member of the bone morphogenetic protein antagonist family, has been implicated in cell proliferation and survival. However, GREM1’s role in cell aging and senescence has never been investigated in human cardiac mesenchymal progenitor cells (hMPCs). Therefore, this study assessed the hypothesis that overexpression of GREM1 rejuvenates the cardiac regenerative potential of aging hMPCs to a youthful stage and therefore allows better capacity for myocardial repair. We recently reported that a subpopulation of hMPCs with low mitochondrial membrane potential can be sorted from right atrial appendage-derived cells in patients with cardiomyopathy and exhibit cardiac reparative capacity in a mouse model of myocardial infarction. In this study, lentiviral particles were used to overexpress GREM1 in these hMPCs. Protein and mRNA expression were assessed through Western blot and RT-qPCR. FACS analysis for Annexin V/PI staining and lactate dehydrogenase assay were used to assess cell survival. It was observed that cell aging and cell senescence led to a decrease in GREM1 expression. In addition, overexpression of GREM1 led to a decrease in expression of senescence genes. Overexpression of GREM1 led to no significant change in cell proliferation. However, GREM1 appeared to have an anti-apoptotic effect, with an increase in survival and decrease in cytotoxicity evident in GREM1-overexpressing hMPCs. Overexpressing GREM1 also induced cytoprotective properties by decreasing reactive oxidative species and mitochondrial membrane potential. This result was associated with increased expression of antioxidant proteins, such as SOD1 and catalase, and activation of the ERK/NRF2 survival signal pathway. Inhibition of ERK led to a decrease in GREM1-mediated rejuvenation in terms of cell survival, which suggests that an ERK-dependent pathway may be involved. Taken altogether, these results indicate that overexpression of GREM1 can allow aging hMPCs to adopt a more robust phenotype with improved survival capacity, which is associated with an activated ERK/NRF2 antioxidant signal pathway.

## 1. Introduction

Cardiovascular disease (CVD) is the leading cause of mortality in the United States [1]. One of the main contributors to CVD is ischemic heart disease, which includes myocardial infarction and post-infarct heart failure [2]. The largest proportion of deaths due to ischemic heart disease occur in elderly patients, over 65 years of age, following myocardial infarction [3]. A promising strategy to regenerate the damaged myocardium in post-infarct patients is progenitor cell-based cardiac therapy. The idea behind cell therapy for myocardial regeneration is to transplant progenitor cells that provide a sustainable source of proliferating, functional cardiomyocytes and lead to repair via direct and indirect mechanisms, including promotion of neovascularization, favorable modulation of the extracellular matrix, and inhibition of apoptosis [4,5]. Cardiac progenitor cells represent a new strategy for the treatment of ischemic cardiomyopathy in the older population [6,7,8]. So far, there are many different populations of human cardiac stem/progenitor cells that have been examined, with the majority of cells sorted according to the differential expression of cell surface markers. We recently isolated a subpopulation of progenitor cells based on low mitochondria membrane potential, named them as human cardiac mesenchymal progenitor cells (hMPCs), and demonstrated their potential for improving myocardial repair [9].

The goal of cell therapy for ischemic heart disease is to deliver the cells to the ischemic area and enable the production and secretion of pro-survival, anti-inflammatory, and regenerative growth factors, which, in turn, will activate survival signaling pathways in the cells [10]. The main obstacle to the success of clinical cell therapies is the age-related loss of progenitor cell regenerative capacity. Cells from older patients have been shown to have diminished proliferative and differentiation potential [11]. Over 90% of transplanted mesenchymal stem cells disappeared within the initial few days after transplantation [12]. Aging leads to the accumulation of damage because of increased oxidative stress, mitochondrial dysfunction, and genome instability [13]. Accumulation of damage and shortening of telomeres leads to cellular senescence, defined as a state of irreversible growth arrest [14]. Cellular senescence is a biological process that occurs after a cell has undergone a finite number of divisions and has lost the ability to replicate. Overall, senescent cells are defined by a low capacity to contribute to cardiac repair and regeneration [15]. Furthermore, in the heart, aging is also associated with reactive oxygen species (ROS) that are generated by the mitochondria [16,17]. Overproduction of ROS in mitochondria also has been associated with cellular senescence; it leads to the formation of reactive products O_2_^−^ or H_2_O_2_, whose accretion accelerates senescence, DNA mutations, inflammation, and cell death pathways [13]. Overall, there are many obstacles associated with the aging progenitor cells; rejuvenation of these aging progenitor cells is necessary in order to improve the efficacy of progenitor cell-based cardiac therapy [18].

A variety of pathways involved in cell survival, proliferation, and senescence can be targeted for rejuvenation through genetic modification. Gremlin1 (GREM1) is a potential target, as it has recently been shown to play a role in the self-renewal of stem cells. GREM1-expressing stem cells were shown to have a capability for self-renewal, multipotentiality, and continued functionality following transplantation [19]. Gremlin1 (GREM1), a secreted glycoprotein, is a member of the cysteine knot superfamily that includes transforming growth factor-β (TGF-β) and vascular endothelial growth factor (VEGF) [20]. Gremlins have diverse effects on several pathways including sonic hedgehog (SHH), fibroblast growth factor (FGF), Wnt and Notch ligand expression and signaling, cell cycle control proteins, and cytokine signaling [21]. GREM1 antagonizes bone morphogenetic proteins (BMPs) by preventing these ligands from interacting with their receptors [22]. GREM1 has been implicated as a novel pro-angiogenic factor that promotes angiogenesis in a BMP-independent manner by enhancing vascular endothelial growth factor receptor-2 (VEGFR2)-dependent angiogenic responses [20,23].

Additionally, GREM1 has been shown to regulate myogenic progenitor proliferation [24]. Furthermore, GREM1 has been implicated in fine modulation of hematopoietic progenitors and lineage decisions in the stem niche in vivo by playing a role in the Notch and hedgehog signaling pathways [25]. GREM1 is also involved in cardiomyocyte differentiation; it was found to enhance the determined path to cardiomyogenesis in a stage-specific manner [26]. Additionally, a recent study has examined the beneficial effects of GREM1 on mesenchymal stem cell (MSC) based therapy for hindlimb ischemia. The study showed that GREM1 overexpression led to increased survival of MSCs exposed to oxidative stress. In addition, MSCs modified with GREM1 significantly enhanced the blood perfusion of the ischemic hindlimb in an in vivo mouse model [27].

Overall, the literature associates GREM1 with regulation of cell growth, survival, and differentiation. However, the relationship between GREM1 and cell senescence and cell aging has not yet been determined. Furthermore, the role of GREM1 has never been investigated in hMPCs. Therefore, in this study, overexpression of GREM1 was used to rejuvenate aging hMPCs and improve cardiac regenerative potential. We hypothesized that overexpression of GREM1 rejuvenates the cardiac regenerative potential of aging hMPCs to a youthful stage and improves their capability for effective cell therapy.

We establish for the first time that GREM1 protein levels are associated with cell aging and cell senescence in hMPCs. We also provide evidence that overexpression of GREM1 has a cytoprotective effect, with improvement in cell survival and increased expression of anti-apoptotic proteins. Furthermore, we show that overexpression of GREM1 decreases ROS and mitochondrial membrane potential, along with increasing antioxidant proteins and activating the ERK/NRF2 survival pathway. These findings suggest that GREM1 can compel aging hMPCs to adopt a more robust phenotype, and that GREM1 is a potential therapeutic target for improving the efficacy of cardiac mesenchymal progenitor cell therapy for ischemic heart disease in the future.

## 2. Materials and Methods

### 2.1. Reagents

Collagenase II was purchased from Worthington Biochemical. Ham’s F12 medium was ordered from Invitrogen (Waltham, MA, USA). Fetal bovine serum (FBS) was obtained from Hyclone. Quantitative PCR primers for target genes were obtained from IDT and listed in the Supplemental Table S1. The primary antibodies are listed in the Supplemental Table S2. Unless indicated otherwise, chemicals used in experiments were ordered from Sigma.

### 2.2. Human Heart Tissue Collection and Δψ_m_^low^-hMPCs’ Isolation

The protocol for collecting human heart samples in the present study was approved by the Institutional Committee on Research at Albany Medical College (IRB#3728), and written informed consent was signed by the patients. hMPCs were isolated from right atrial appendage-derived cells from patients with ischemic cardiomyopathy, as described previously [9]. Adult/aging hMPCs were derived from patients who were 52 years of age or older. Young/pediatric hMPCs were sorted from cells derived from right atrial or atrial septum (see Table 1). Briefly, myocardial tissues were minced into small pieces and enzymatically digested with collagenase II (30 units/mL) with gentle shaking at 37 °C for 1 h. After incubation on ice for 10 min, the undigested clumps were separated by gravity. Supernatant was moved into a 15 mL tube and centrifuged at 1400 rpm for 5 min. The resulting cell pellet was resuspended and cultured in hMPC growth medium. hMPCs were sorted using flow cytometry, based on low mitochondrial membrane potential, using 25 nM of the potentiometric dye tetramethylrhodamine methyl ester (TMRM) for 30 min at 37 °C. Cells with the lowest 3% TMRM fluorescence intensity were collected and used for subsequent studies. These cells have been characterized as positive (>90%) for the surface markers CD172a, CD140b, CD90, and CD44, partially positive for the surface marker CD172b (~30%), but negative (<5%) for the surface markers CD34, CD45, CD54, CD117 (c-kit), and CD133 [9].

### 2.3. Cell Culture

hMPCs were cultured in the defined growth medium containing Ham’s F-12 (Invitrogen, Waltham, MA, USA), 10% FBS (Hyclone, Logan, UK), 10 ng/mL human basic FGF (fibroblast growth factor) (Sigma, St. Louis, MA, USA), 0.005 unit/mL human EPO (erythropoietin) (Sigma), 0.2 mM L-glutathione (Sigma), and 100 units/mL penicillin and 100 g/mL streptomycin (Gibco, Grand Island, NY, USA). All cells were cultured in the condition with 5% CO_2_ and 5% O_2_.

### 2.4. Lentiviral Production and Infection of hMPCs

The bacterial stock of ORF expression clone (Catalog# EX-W0207-Lv105) for GREM1 (GeneCopoeia, Rockville, MD, USA) was used to grow bacterial colonies. After 12–16 h of growth, a single bacterial colony was picked from the agar dish and grown in liquid Luria broth (LB) culture, with vigorous shaking for 12–16 h. Maxiprep was performed using Qiagen kit to obtain plasmid DNA. The quality and quantity of DNA was detected by a Nanodrop 2000C spectrophotometer (Thermo Scientific, Waltham, MA, USA). HEK293FT (human embryonic kidney) packaging cells were seeded in T-75 flasks 24 h prior to transfection. GREM1 plasmid DNA was co-transfected into 70% confluent HEK293FT packaging cells along with 2 packaging plasmids, psPAX2 and pMD2.G, using the Gene Jammer reagent (Agile Technologies, Aliso Viejo, CA, USA), with a plasmid:psPAX2:pMD2.G ratio of 4:3:1. Medium was replaced with normal growth medium for recovery 18–24 h after transfection. The supernatant from the transfected cells was collected 48 h and 72 h after transfection. Lenti-X GoStix (Takara, Shiga, Japan) was used to confirm the presence of lentivirus. The Lenti-X Concentrator (Takara) was used to concentrate lentiviral stocks 25×.

Specific overexpression of GREM1 in hMPCs was carried out using lentiviral particles. Vector lentiviral particles were used as a control. hMPCs were seeded into a 6-well plate for 24 h before infection. 24 h later, with the cells around 70% confluency, medium was refreshed via direct addition of 5 µg/mL Polybrene and appropriate concentration of lentiviral particles and shaken gently a few times. For further culturing, medium was replaced by the fresh growth medium 18–24 h after infection.

### 2.5. ERK Inhibition

Cells were seeded and infected with lentiviral particles normally, as described above. The media was refreshed with 10 µM of the ERK inhibitor U0126 18–24 h after infection. U0126 was applied to the hMPCs for 24 h. U0126 was demonstrated to inhibit ERK activity in human cardiac progenitor cells in our previous publication [28], the efficacy of which was confirmed in hMPCs by Western blotting.

### 2.6. Cell Viability Assay

LDH release assay, a well-known indicator of cell membrane integrity and viability, was used to examine the cell death induced by oxidative stress. The detailed protocol was followed based on the manufacturer’s instructions provided in the LDH detection kit (Takara). Between 36 and 48 h after infection with lentiviral particles, oxidative stress was induced by exposing the cells to 2 mM H_2_O_2_ for 3 h, as optimized based on previous studies [28,29]. An amount of 100 μL of the supernatant was collected, centrifuged at 250× *g* for 5 min, and mixed with an equal volume of pre-prepared solution (catalyst/dye 1:45) for 30 min at room temperature in a 96-well plate. The absorbance of samples at 490 nm was measured using a Bio-Rad iMark microplate reader. The percentage of LDH release for each sample was normalized according to the absorbance reading from low-control samples that were treated with plain F-12 serum-free media and high-control samples that were treated with 0.5% Triton X-100.

### 2.7. Senescence-Associated Beta-Galactosidase Assay

Senescence-associated beta-gal staining kit (Cell Signaling Technology, Inc., Danvers, MA, USA) was used to qualitatively measure senescence. After lentiviral infection to overexpress GREM1 for 48 h, hMPCs were washed 2 times with PBS and fixed with the solution provided according to manufacturer’s instructions. After adding the beta-galactosidase staining solution, cells were incubated in the plate at 37 °C for 24 h. Beta-gal-positive cells were identified by counting cells with blue color using microscope.

### 2.8. FACS Analysis for Apoptotic Assay

The percentage of apoptotic, necrotic, and viable cells in the different groups was determined with annexin V/propidium iodide (PI) apoptosis detection kit (Invitrogen) with dual staining. Between 36 and 48 h after infection with lentiviral particles, hMPCs were trypsinized and then resuspended in 1 mM H_2_O_2_ in serum-free F-12 medium. Here, we chose a relatively moderate condition (1 mM dose of H_2_O_2_ for 90 min) for the apoptotic assay, where we expected to induce early and late cell apoptosis. The condition (2 mM H_2_O_2_ for 3 h) used in the LDH assay can cause massive cell necrosis, which is not a good condition for the apoptotic assay with the annexin V/PI staining. After 90 min of exposure to H_2_O_2_, the cells were washed in PBS, and resuspended in 50 µL of annexin-binding buffer supplemented with 1 µg/mL PI and 1 µL of Alexa Flour 647-annexin V solution (Invitrogen). After 15 min of incubation, 100 µL of 1× annexin-binding buffer was added with gentle shaking, and samples were analyzed by flow cytometry with Guava EasyCyte System (EMD Millipore Corporation, Burlington, MA, USA) [28,29]. Data was quantified by GuavaSoft Module software. Cells positive for annexin V only (Q4), or double positive for both annexin V and PI (Q2), were defined as apoptotic cells. Cells positive for PI only (Q1) were defined as necrotic cells, while cells negative for both annexin V and PI (Q3) were defined as cells that had survived. All quadrants are labeled on the representative FACS images.

### 2.9. Cell Proliferation Assay with BrdU Incorporation

hMPCs positive for BrdU were analyzed by flow cytometry to examine the effect on cell proliferation capability, as described previously [28,29]. In short, 18–24 h after lentiviral infection, the growth medium was refreshed and supplemented with 10 µM BrdU. Then, 36–48 h after infecting, the cells were detached by trypsinization and fixed with 4% paraformaldehyde. Cells were treated with 2 M HCl for 30 min and permeabilized with 0.5% Triton X-100 for 20 min. After sufficient PBS washes, cells were resuspended in blocking buffer (2% BSA in PBS) for 30 min, subsequently stained with BrdU antibody in blocking buffer for 2 h at room temperature, and then incubated with a secondary antibody (Alexa Fluor 647 Goat anti-Mouse; Life Technologies, Carlsbad, CA, USA) for 1 h at room temperature. Cells were subsequently diluted in PBS for flow cytometry analysis.

### 2.10. Measurement of ROS and Mitochondrial Membrane Potential

Dihydroeithidium (DHE) cellular ROS detection assay kit was used to measure ROS [30]. The cells were collected by trypsinization 36–48 h after infection with lentiviral particles, washed with PBS, and subsequently stained with 10 µM DHE dye for 30 min at 37 degrees in the dark. The intensity of red-blue fluorescence was examined by Guava EasyCyte System and subsequently analyzed.

Mitochondrial membrane potential was measured via staining with tetramethylrhodamine, methyl ester (TMRM), a cell-permeant dye that uptakes in active mitochondria with intact membrane potentials. Between 36 and 48 h after infection with lentiviral particles, cells were collected by trypsinization, washed with PBS, and subsequently stained with 25 nM TMRM dye (Sigma) for 30 min at 37 degrees. The intensity of yellow-blue fluorescence was detected by flow cytometry.

### 2.11. RNA Isolation and Quantitative Analysis

The total RNA was extracted and isolated using the Aurum Total RNA minikit (Bio-Rad, Hercules, CA, USA). The quality and quantity of RNA was detected by a Nanodrop 2000C spectrophotometer (Thermo Scientific). The reverse transcription of RNA to cDNA was performed using the Bio-Rad iScript cDNA synthesis kit. Samples for real time PCR were produced according to the manufacturer’s instructions with primers from IDT in the iQSYBR Green Supermix kit (Bio-Rad) and real time PCR was run with a BioRad iQ5 optical module. Cyclic conditions of 5 °C for 2 min as initial denaturation, 40 cycles of denaturation at 95 °C for 15 s, and annealing/extension at 60 °C for 40 s were used. In these experiments, the gene glyceraldehyde-3-phosphate dehydrogenase (GAPDH) was used as an internal control gene for quantitative analysis. Supplemental Table S1 lists the primers for genes used in this project.

### 2.12. Immunoblotting

Western blot analysis was conducted according to protocol, as described previously [29,31]. After 2 ice-cold PBS washes, cells were harvested and lysed with ice-cold modified immunoprecipitation assay buffer (150 mM NaCl, 5 mM EDTA, 1% Nonidet P-40, 20 mM Tris-HCl, pH 7.5) with addition of protease and phosphatase inhibitor mixtures (Sigma). Between 15 and 20 µg of protein from each sample was differentiated on 12% SDS-polyacrylamide gels and transferred to nitrocellulose membranes. After incubating with 5% milk in TBST (50 mM Tris-HCl, pH 7.4, 150 mM NaCl, 0.1% Tween 20) buffer for 1 h, primary antibodies against specific genes of interest were applied overnight, and then incubated with HRP-conjugated secondary antibodies for 1.5 h (1:4000 dilutions of anti-rabbit or anti-mouse antibodies; Cell Signaling). The chemiluminescence signals were detected using SuperSignal West Femto Maximum Sensitivity Substrate (Thermo Scientific). The Image Quant LAS 3000 system was used for imaging. GAPDH was used as an equal loading control. Image J software was used to quantify the band density. Please see Supplemental Table S2 for the list of antibodies used in this project.

### 2.13. Statistical Analysis

Data were shown as means ± SEM of results from at least three independent repeated experiments. Statistical significance was calculated by Student’s *t*-test for two group comparison, or one-way ANOVA for multiple group comparison. A *p*-value less than 0.05 was considered as statistically significant.

## 3. Results

### 3.1. GREM1 Expression Is Associated with Cell Aging and Cell Senescence

We have previously established a protocol to isolate hMPCs with low mitochondrial membrane potential from human cardiac cells derived from atrial appendages in patients [9]. With this protocol, we were able to sort hMPCs from patients of different ages. In the present study, we randomly chose 4 lines of pediatric hMPC sorted from atrial-derived cells in patients aged 2–15 years old. Accordingly, 4 lines of adult/aging hMPCs from atrial-derived cells at the ages of 52–84 years old were chosen for the molecular and biochemical studies below. The patients’ basic demographic information is provided in Table 1.

**Figure 1:**
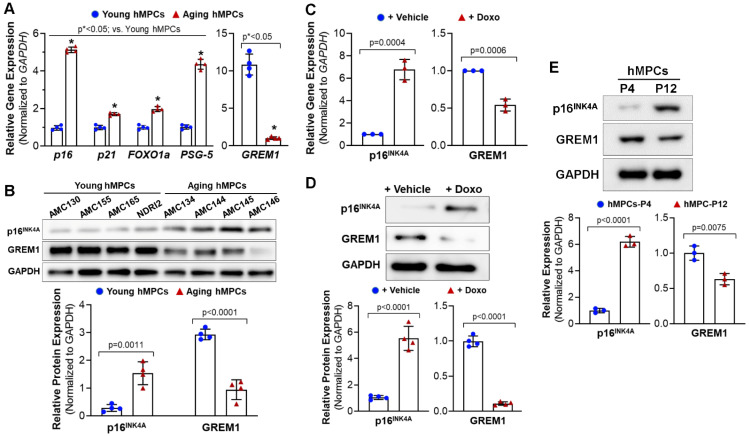
Decreased expression of GREM1 is associated with the aging/senescence of cardiac mesenchymal progenitor cells (hMPCs). (**A**) Gene expression of cell senescent markers (p16, p21, FOXO1a, and PSG5, as well as GREM1) in four lines of pediatric and aging hMPCs. (**B**) Western blot analysis for the protein expression of p^16INK4A^ and GREM1 in four lines of pediatric and aging hMPCs. (**C**) Gene expression of p16^INK4A^ and GREM1 in the pediatric hMPCs with 12 h doxorubicin-induced cell senescence. (**D**) Western blot analysis for the protein expression of p16^INK4A^ and GREM1 in the pediatric hMPCs with 12 h doxorubicin-induced cell senescence. (**E**) Western blot analysis for the protein expression of p16^INK4A^ and GREM1 in the pediatric hMPCs with different passage number following replicative senescence. Data are mean ± SEM. *p* value was calculated by unpaired Student’s *t*-test, *n* ≥ 3 independent experiments. * indicated the significant difference compared to the control group.

Previous studies have indicated that GREM1 is associated with cell survival and proliferation; however, no study to date has assessed GREM1’s role in cell aging and senescence. To determine the effect of cell aging on GREM1 expression, real time quantitative polymerase chain reaction (RT-qPCR) was conducted for the RNA samples isolated from the four lines of pediatric hMPCs and four lines of adult/aging hMPCs. As shown in Figure 1A (*left panel*), adult/aging hMPCs exhibited greater gene expression of senescence and quiescence markers, such as p16, p21, FOXO1a, and NANOG, compared to pediatric hMPCs. Meanwhile, the gene expression of GREM1 was significantly lower in aging hMPCs than in pediatric hMPCs (Figure 1A, *right panel*).

Data obtained from Western blots confirmed the increased p16^INK4A^ and decreased GREM1 protein expression in aging hMPCs (Figure 1B) as compared to pediatric hMPCs, suggesting that genetic modification of GREM1 expression may allow for rejuvenation of aging hMPCs and coerce them to adopt a more resilient phenotype, similar to that of pediatric hMPCs.

Cell senescence is one of the factors that contributes to aging. Therefore, we assessed GREM1’s role in cell senescence. Doxorubicin is an anticancer drug that has been shown to induce senescence in cells through the production of oxidant species [32]. Doxorubicin has been associated with high levels of ROS that activate cytotoxic signaling leading to DNA damage and mitochondrial dysfunction [33], therefore inducing cell senescence. In this study, cell senescence was induced in hMPCs through treatment with 0.5 µM doxorubicin for 24 h. Successful induction of senescence was confirmed through RT-qPCR for p16, a senescence-associated marker gene. We observed that inducing senescence led to a 6-fold increase in p16 gene expression (Figure 1C, *left panel*). Interestingly, inducing senescence in pediatric hMPCs led to a decrease in GREM1 mRNA (Figure 1C, *right panel*). Results from the Western blots confirmed the increased p16^INK4A^ and decreased GREM1 protein expression (Figure 1D). Normal somatic cells inevitably experience replicative stress and senescence during proliferation. To examine the expression of GREM1 in replicative senescence, Western blots were conducted for pediatric cell lines at early passage (P4) and late passage (P12) following a long-term culture. As shown in Figure 1E, a significant increased protein expression of p16^INK4A^ and decreased protein expression of GREM1 was observed in hMPCs with replicative cell senescence (P4) compared to its same line of hMPCs at early passage. Taken together, these results suggest the decreased expression of GREM1 is strongly associated with the aging of senescence of hMPCs.

### 3.2. Overexpression of GREM1 Reverses the Senescent Phenotype of hMPCs

**Figure 2:**
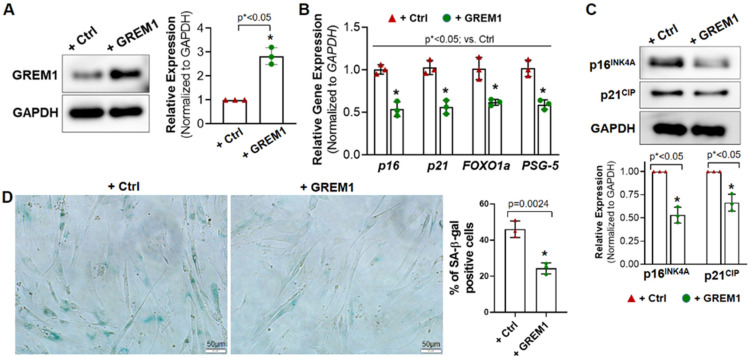
Overexpressing GREM1 rejuvenates the senescent phenotype of aged hMPCs. (**A**) Western blot analysis confirmed the overexpression of GREM1 after infecting hMPCs with lenti-GREM virus or control (Ctrl) lentivirus. *n* = 3 independent experiments. (**B**) qPCR for the gene expression of p16, p21, Foxo1a, and PSG-5 in aged hMPCs (AMC145) after overexpressing GREM1. *n* = 3 independent experiments. (**C**) Western blot analysis showed the protein level of p16 and p21 in aged hMPCs (AMC145) after overexpressing GREM1. *n* = 3 independent experiments. (**D**) Representative images and quantitative analysis of SA-β-gal staining in aged hMPCs (AMC145) after overexpressing GREM1. Data are mean ± SEM. *p* value was calculated by unpaired Student’s *t*-test, *n* ≥ 3 independent experiments. * indicated the significant difference compared to the control group.

The association of GREM1 expression with cell aging and cell senescence indicated its potential ability to rejuvenate aging hMPCs. Therefore, we generated lentiviral particles to overexpress GREM1 in hMPCs. Western blot analysis showed that lentiviral-mediated overexpression of GREM1 resulted in a 2.5-fold increase in GREM1 protein expression (Figure 2A) in all 3 aging lines of hMPCs (AMC144, AMC145, AMC146). Overexpression of GREM1 in aging hMPCs also led to a change in expression of several senescence and quiescence markers. Expression of p16, p21, FOXO1a, and PSG-5 was found to decrease in GREM1-overexpressed hMPCs as compared to vector-overexpressed hMPCs, as seen by RT-qPCR (Figure 2B). In addition, results from Western blot analysis showed that the protein level of p16^INK4A^ and p21^CIP^ was significantly reduced upon overexpressing GREM1 in hMPCs compared to the control cells (Figure 2C). Interestingly, senescence-associated (SA) beta-gal staining showed decreased numbers of senescent cells in the experimental hMPCs infected with lentivirus-expressing GREM1 (Figure 2D) compared to the control hMPCs. Altogether, the data suggest that overexpression of GREM1 can suppress cell senescence, as evidenced by the decreased expression of markers for senescence.

GREM1 has previously been shown to play a role in cell proliferation in other tissues of the body [34]. Therefore, to evaluate the effect of GREM1 overexpression on hMPCs’ proliferation ability, we performed a BrdU cell proliferation assay. However, the BrdU incorporation experiment showed no significant difference in BrdU+ cells between GREM1-overexpressed and vector-overexpressed hMPCs (Supplemental Figure S1), indicating that overexpressing GREM1 does not change the proliferation of hMPCs.

### 3.3. Overexpression of GREM1 Exhibits an Anti-Apoptotic Effect in Aging hMPCs

**Figure 3:**
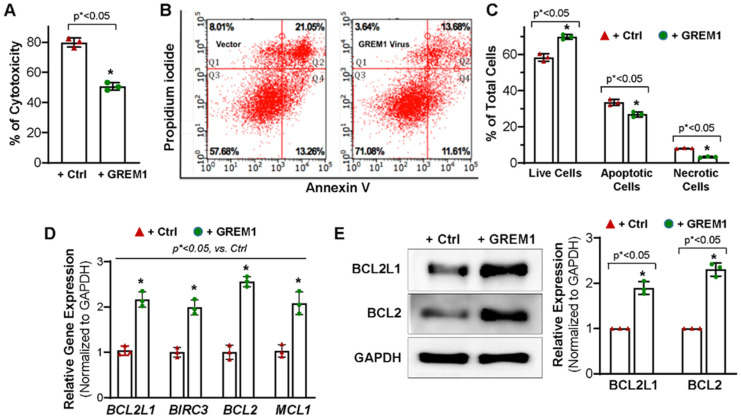
Overexpressing GREM1 enhances the survival capacity of the hMPCs and increases the expression of anti-apoptotic genes and protein. (**A**) Overexpressing GREM1 in hMPCs significantly increased the cell survival capacity, as indicated by the decreased LDH release. (**B**) FACS analysis with annexin V/PI staining shows a significant increase in the number of live cells (Q3) and a decrease in the number of apoptotic cells (Q2 + Q4) and necrotic cells (Q1) after overexpressing GREM1 in hMPCs compared to the control cells. (**C**) Quantitative analysis for panel B. (**D**) Real-time quantitative PCR analysis with an anti-apoptotic primer library screening shows a significant increase of the indicated anti-apoptotic gene expression following 48 h of overexpressing GREM1 in hMPCs versus control cells. (**E**) Western blot analysis showed the protein level of BCL2L and BCL2 in hMPCs with overexpressing GREM1 versus control cells. Data are mean ± SEM. *p* value was calculated by unpaired Student’s *t*-test, *n* = 3 independent experiments. * indicated the significant difference compared to the control group.

A previous study showed that GREM1-overexpressing MSCs reduced the amount of apoptosis induced by oxidative injury [27]. To assess the effect of GREM1 overexpression on cell viability, hMPCs were incubated with 2 mM H_2_O_2_ for 3 h. Cytotoxicity was evaluated using an LDH release assay. There was a 30% decrease in cytotoxicity evident upon overexpression of GREM1 (Figure 3A).

To further evaluate the role of GREM1 in promoting survival in hMPCs, annexin V/PI staining was performed followed by flow cytometry analysis. Oxidative stress was induced by treatment with 1 mM H_2_O_2_ for 90 min. FACS analysis showed live cells with double negative staining in quadrant 3 (Q3). Early apoptotic cells with only annexin V staining were seen in quadrant 4 (Q4), whereas cells with dually positive annexin V and PI staining represented cells in late apoptosis and were seen in quadrant 2 (Q2). Cells in quadrant 1 represented PI positive necrotic cells (Figure 3B). Overexpression of GREM1 led to a significant increase in the total number of live cells (69.8 ± 1.1%) compared to cells expressing vector (58.4 ± 1.2%). Meanwhile, the number of apoptotic cells significantly decreased between vector control hMPCs (33.5 ± 2.78%) and GREM1-overexpressing hMPCs (26.9% ± 1.5%). In addition, the GREM1-overexpressing population of hMPCs had a significantly lower percentage of necrotic cells (3.23 ± 0.37%) as compared to vector control (8.12 ± 0.14%) (Figure 3C). This was consistent with the results described above for the LDH release assay. Taken together, these results suggested that overexpressing GREM1 enhance the survival capacity of hMPCs against H_2_O_2_-induced oxidative stress.

To evaluate the mechanism by which GREM1 may be regulating cell apoptosis, RT-qPCR for anti-apoptotic genes was conducted for hMPCs with or without GREM1 overexpression. As shown in Figure 3D, overexpression of GREM1 significantly increased the gene expression of BCL2L1 (BCL-xL), BIRC3, BCL2, and MCL1. Results from Western blots using the antibodies against BCL2L1 and BCL2 showed a significant increase in expression of these proteins in GREM1-overexpressing hMPCs (Figure 3E). We also examined the expression of pro-apoptotic proteins, including BAX, BAK, and BID, for hMPCs, and we did not see significant differences for the expression of these pro-apoptotic proteins for hMPCs with or without GREM1 overexpression (data not shown). Taken altogether, the data indicate that GREM1 is a promising target for rejuvenation by genetic modification, and overexpression of GREM1 can lead to a cytoprotective effect in hMPCs.

### 3.4. Overexpression of GREM1 Reduces ROS Generation and Increase the Expression of Antioxidants

**Figure 4:**
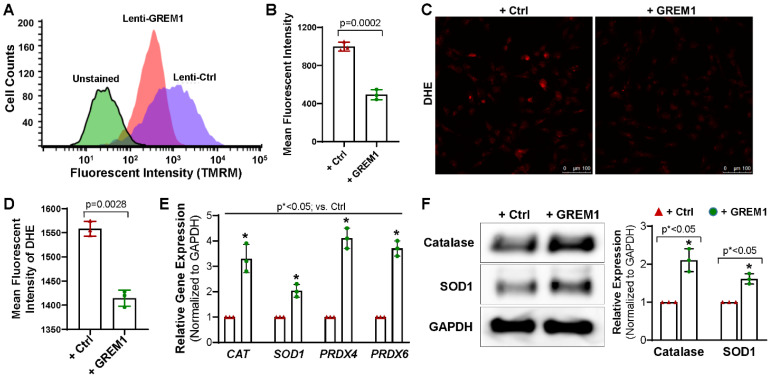
Overexpressing GREM1 decrease the ROS generation in hMPCs and increase the expression of antioxidant proteins. (**A**) Representative merged histogram for mitochondrial ROS measurement for aged hMPCs overexpressing GREM1 vs. vector control by FACS analysis following TMRM live cell staining. (**B**) Quantitative analysis for panel A. (**C**) Representative images for DHE staining for aged hMPCs infected with GREM1 or vector lentivirus for 48 h. (**D**) Quantitative analysis for C. (**E**) Real-time quantitative PCR analysis with an antioxidant primer library screening shows the increased gene expression of CAT, SOD1, PRDX4, and PRDX6 in hMPCs with GREM1 overexpression. (**F**) Representative images and quantitative data of Western blot showing the expression level of antioxidant proteins (catalase and SOD1) after GREM1 was overexpressed. Full-length blots are presented in Supplemental Figure S2. Data are mean ± SEM. *p* value was calculated by unpaired Student’s *t*-test, *n* = 3 independent experiments. * indicated the significant difference compared to the control group.

Next, we examined whether genetic modification of GREM1 leads to an effect on the intracellular redox state of hMPCs. Progenitor cells with lower mitochondrial membrane potential have been described as exhibiting lower ROS. Since cells with lower mitochondrial membrane potential have been shown to be more suitable for cell therapy and in vivo persistence [9,35], we hypothesized that overexpression of GREM1 should decrease mitochondrial membrane potential. Mitochondrial membrane potential was measured by staining live cells with TMRM and subsequently analyzing with flow cytometry. hMPCs that overexpressed GREM1 showed a significant decrease in mitochondrial membrane potential, as shown by the decrease in mean fluorescence intensity for TMRM (Figure 4A,B). A leftward shift in the peak value was also visible between GREM1-overexpressing hMPCs and vector control (Figure 4A).

Additionally, fluorescent microscope imaging was conducted for cultured hMPCs following the staining with dihydroethidium (DHE), a fluorescence indicator for superoxide. An increase in the fluorescence intensity for DHE indicates an increase in oxidation. Overexpression of GREM1 significantly reduced the fluorescent intensity of DHE (Figure 4C,D), suggesting that there is a decrease in ROS in hMPCs upon overexpressing GREM1. Taken together, these results indicate that overexpressing GREM1 can reduce oxidative stress. Accumulation of ROS is one of the hallmarks of aging, and GREM1-induced reduction in ROS is promising for the rejuvenation of aging hMPCs.

To expand on the results described above, we wanted to assess the effect of GREM1 overexpression on the expression of antioxidants. Results from qPCR showed that the expression of antioxidant genes, including *CAT, SOD1, PRDX4*, and *PRDX6*, were considerably increased in hMPCs with GREM1 overexpression compared to the control cells (Figure 4E). Western blots further confirmed the increase of Catalase and SOD1 protein expression in GREM1-overexpressing hMPCs (Figure 4F), suggesting that GREM1 allows for modulation of oxidative stress. Overall, all the data suggest that overexpressing GREM1 has an antioxidant role when overexpressed in hMPCs.

### 3.5. Activation of NRF2/ERK Signal Pathway Is Associated with the Cytoprotective Effect of Overexpression of GREM1

**Figure 5:**
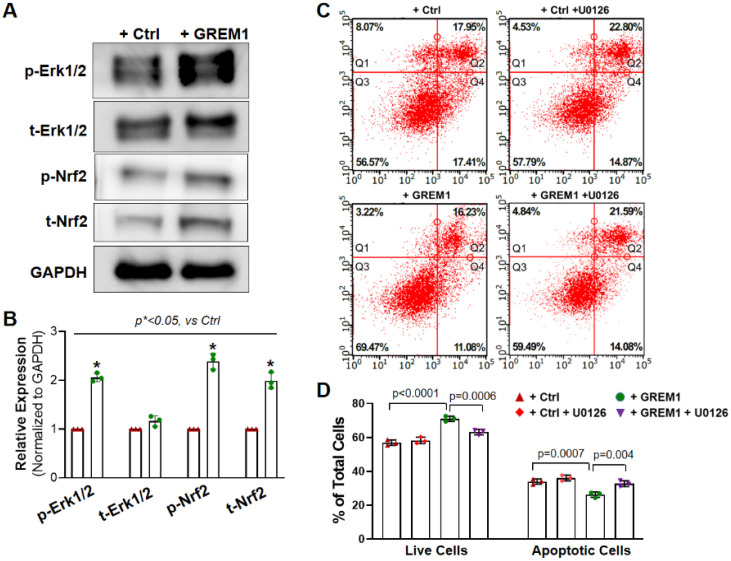
Activation of NRF2/ERK signal pathway is associated with the cytoprotective effect of overexpressing GREM1. (**A**) Representative image of Western blots showing increased protein expression of total and phosphorylated ERK1/2 and NRF2 in hMPCs with GREM1 overexpression. (**B**) Quantitative analysis of panel A. (**C**) FACS analysis with annexin V/PI double staining shows that specific inhibition of ERK activity by U0126 diminishes the cytoprotective effect of overexpressing GREM1 in aged hMPCs, as indicated by the decreased number of live cells (Q3) and increased number of apoptotic cells (Q2 + Q4) compared with cells without U0126 treatment as control. (**D**) Quantitative analysis of the FACS assay shown in C. Data are mean ± SEM. *p* value was calculated by unpaired Students’ *t*-test, *n* = 3 independent experiments. * indicated the significant difference compared to the control group.

ERK/NRF2 is one of the major survival signal pathways that were associated with stem/progenitor cell therapy [28,36]. Earlier studies in our lab have shown that the ERK/NRF2 pathway is associated with the cytoprotective effect of preconditioning human cardiac progenitor cells with CoPP [28]. Furthermore, recent work has shown that the ERK/NRF2 signal pathway is implicated in protecting hMPCs against oxidative stress-induced apoptosis [36]. Therefore, we wanted to investigate whether GREM1’s effects on improving cell survival may also be through the activation of the ERK/NRF2 pathway. We conducted Western blots for phosphorylated ERK1/2 and NRF2 in hMPCs with or without GREM1 overexpression. As shown in Figure 5A,B, both the phosphorylated and total protein level of ERK1/2 and NRF2 were significantly increased in hMPCs with GREM1 overexpression compared to the control cells, suggesting that overexpression of GREM1 can activate the ERK/NRF2 signal pathway.

To further investigate the mechanism behind GREM1-mediated hMPCs’ rejuvenation, we examined the effect of ERK inhibition on oxidative stress-induced apoptosis with FACS analysis following annexin V/PI staining. Since GREM1 overexpression led to upregulation of the ERK/NRF2 signal pathway (Figure 5A), we hypothesized that inhibition of ERK would lead to a reversal in GREM1-mediated rejuvenation, in terms of cell survival and apoptosis. ERK was inhibited through the treatment of hMPCs with 10 µM U0126 for 24 h [28]. Treatment of GREM1-overexpressing hMPCs with the ERK inhibitor U0126 led to a significant decrease in the number of live cells in comparison to cells that overexpressed GREM but were not treated with U0126. There was also a significant increase in cell apoptosis in GREM1-overexpressing hMPCs that were treated with U0126 (Figure 5C,D). Furthermore, after the addition of the inhibitor, there was no longer a statistically significant difference in survival or apoptosis between vector and GREM1 groups such as the one found earlier without the inhibitor (Figure 5C). This data suggests that an ERK-dependent pathway plays a critical role in GREM1-mediated hMPC survival.

## 4. Discussion

Improving the efficacy of stem/progenitor cell therapies is necessary for successful treatment of ischemic heart disease. Obstacles to the success of progenitor cell-based clinical therapies include the poor survival of donor cells along with the age-related loss of cell regenerative capacity. Overall, progenitor cells harbor the intrinsic ability to accelerate healing of damaged tissue, but it is necessary to perform in vitro modification to enhance their regeneration potential. More than 90% of stem cells have been described as dying within the first few days after transplantation [12]. In this study, we show that GREM1 has the capability to improve the survival of progenitor cells. The results show that overexpression of GREM1 leads to a significant increase in cell survival and a decrease in cell apoptosis under the condition of oxidative stress. The data also shows that overexpression of GREM1 activates survival signal pathways, including ERK and NRF2 and, as well as increasing the expression of anti-apoptotic proteins BCL-xL and BCL2, and of antioxidant proteins SOD1 and catalase. The increase in antioxidant proteins is consistent with results from flow cytometry, which showed a decrease in ROS and a decrease in mitochondrial membrane potential in GREM1-overexpressing hMPCs. This study is significant because modulating the expression of GREM1 can be used to promote the regenerative potential of progenitor cells and improve the efficacy of cell therapy for ischemic heart disease.

GREM1 is a gene that is fundamentally required for normal development as an inhibitor of BMP [37]. It is involved in limb patterning and kidney morphogenesis [38]. However, apart from its role in development, it has effects that are independent of BMP action. GREM1 is a pro-angiogenic factor expressed by endothelial cells; its angiogenic effects are mediated via VEGF [20]. In addition, a recent study has shown that overexpression of GREM1 protected MSCs from oxidative injury. Furthermore, in that study, they also showed that transplantation of GREM1-MSCs improved the survival of the transplanted cells and the resident cells in the ischemic environment [27]. However, it remains unclear whether GREM1 plays a cytoprotective role in human cardiac progenitor cells. Therefore, our study assessed GREM1’s role in hMPCs, a new subpopulation of human cardiac progenitor cells that we recently reported [9]. Our study was also the first to correlate GREM1 expression with cell aging and senescence, the major barriers of cell transplantation. Our results depict a correlation between GREM1 expression and cell aging and cell senescence. Overexpression of GREM1 led to a decrease in senescence genes. Since senescent cells lack the ability to contribute to tissue repair and regeneration, the decrease in senescence seen with GREM1 overexpression may allow hMPCs to be able to better aid in tissue repair when transplanted after myocardial infarction. Because the heart comprises a heterogeneity of cells, including cardiomyocytes, fibroblasts, endothelial cells, smooth muscle cells, neuronal cells, and macrophages, all of these cells might modulate the phenotype of the hMPCs through their paracrine activities. It is critical to confirm whether overexpression of GREM1 in hMPCs can reverse the aging and senescence phenotypes using an in vivo system. We plan to conduct in vivo studies on the transplantation of GREM1-overexpressed hMPCs into immuno-deficient NOD scid gamma (NSG) mouse hearts soon, and hope to publish the results in a separate manuscript in the future.

Several studies have highlighted the role of the GREM family of proteins in promoting cell proliferation [39]. Overexpression of GREM1 has been shown to increase vascular smooth muscle cell proliferation and migration [40]. However, other studies have shown that GREM1 can inhibit proliferation; GREM1 was found to inhibit proliferation of myogenic progenitors in human fetal skeletal muscle in a BMP-dependent manner [24]. The conflicting findings from the literature suggest a cell type-specific role for GREM1 in the regulation of cell proliferation [41]. Our results show no significant difference in cell proliferation, as assessed through BrdU^+^ cells between GREM1-overexpressing hMPCs and vector control cells.

GREM1 has been shown to regulate cell apoptosis via the PI3K/Akt pathway in HUVEC cells [27]. Our results suggest that GREM1 regulates cell apoptosis in hMPCs via the ERK/NRF2 signal pathway, as evidenced by the increase in total and phosphorylated ERK and NRF2 in GREM1-overexpressing hMPCs. Inhibition of ERK with U0126 led to an increase in apoptosis in GREM1-overexpressing hMPCs. This suggests that GREM1’s effects on cell survival may be mediated through the ERK pathway. The results indicate that inhibition of ERK can abrogate GREM1’s effects of improving survival and decreasing apoptosis; after the addition of the ERK inhibitor, there was no longer a statistically significant difference in survival or apoptosis between vector and GREM1 groups. Since ERK is associated with cell survival, its inhibition is expected to decrease survival. However, there was no statistical difference in survival and apoptosis between the vector and vector + ERK inhibitor groups. In addition, a complete reversal of GREM1’s effects was not seen, indicating other pathways may also be involved in the ERK pathway to modulate GREM1’s effects.

Cellular redox state and ROS play a major role in regulation of hMPCs’ survival, self-renewal, and differentiation [42]. The production of ATP within the mitochondria through oxidative phosphorylation can lead to the release of harmful ROS as a byproduct. ROS can react with cellular components and contribute to the physiological effects of aging. Our results show that overexpression of GREM1 can lower the level of ROS. Since stem cells with lower ROS levels have been described to engraft better in recipients than cells with higher ROS content [43], our data suggest that hMPCs with overexpression of GREM1 may be more successful after transplantation into the post-infarct heart. The generation of ROS is tightly regulated by scavenging systems, which are comprised of antioxidant enzymes that can neutralize ROS. These antioxidants include SOD, catalase, peroxiredoxins, and glutathione peroxidase [17]. Overexpression of GREM1 showed an increase in these ROS scavenging systems, which is consistent with the lower ROS levels seen with GREM1 overexpression.

Higher mitochondrial membrane potential has been implicated as an indicator of increased electron transport chain activity, which leads to production of ROS [17]. Furthermore, low mitochondrial membrane potential has been associated with long term in vivo persistence and better cell engraftment [35]. Our results indicated that GREM1 overexpression leads to a significant decrease in mitochondrial membrane potential, suggesting that GREM1-overexpressing hMPCs may improve the efficacy of stem cell transplantation for ischemic heart disease.

## 5. Conclusions

Overall, this is the first study to address the role of GREM1 in rejuvenating aging hMPCs toward the goal of promoting cardiac regenerative capacity. We demonstrate that overexpression of GREM1 has an anti-apoptotic and antioxidant effect on aging hMPCs. In addition, the results suggest the involvement of the ERK/NRF2 signal pathway in GREM1’s mediation of cell survival. Future studies are required to examine the in vivo survival capacity of hMPCs after overexpression of GREM1 and determine whether it can improve cardiac structure and function.

## Figures and Tables

**Table 1:** Patients’ basic demographics for established pediatric and adult hMPCs lines used in this study.

Patient Code No.	Gender	Age (Years)	Disease	Tissue Used for Cell Sorting
AMC130	Male	2	ASD	Atrial septum
AMC155	Male	4	ASD	Atrial septum
AMC165	Female	8	ASD	Atrial septum
NDRI2	Female	15	Accident	Right atrial
AMC134	Male	65	CAD	Right atrial appendage
AMC144	Male	52	CAD	Right atrial appendage
AMC145	Female	81	CAD	Right atrial appendage
AMC146	Male	84	CAD	Right atrial appendage

Abbreviation: ASD: atrial septal defect; CAD: coronary artery disease.

## Data Availability

Not applicable.

## Supplementary Materials

The following supporting information can be downloaded at: https://www.mdpi.com/article/10.3390/cells12081203/s1, Figure S1: Overexpression of GREM1 in hMPCs does not alter the cells’ ability to proliferate; Figure S2: Full-length Western blot images for Figure 1B,D,E, Figure 2A,C, Figure 4E and Figure 5A; Table S1: List of primers for human genes used for the qPCR; Table S2: List of antibodies against human proteins used for the Western blots.
