# Supplementary material for: Overexpression of GREM1 Improves the Survival Capacity of Aged Cardiac Mesenchymal Progenitor Cells via Upregulation of the ERK/NRF2-Associated Antioxidant Signal Pathway
Source: Cells. 2023 Apr 21;12(8):1203. doi: 10.3390/cells12081203 (PMC10136744; doi:10.3390/cells12081203)
Supplement: Supplementary file 1 [file cells-12-01203-s001.zip › cells-2311223-supplementary.pdf]

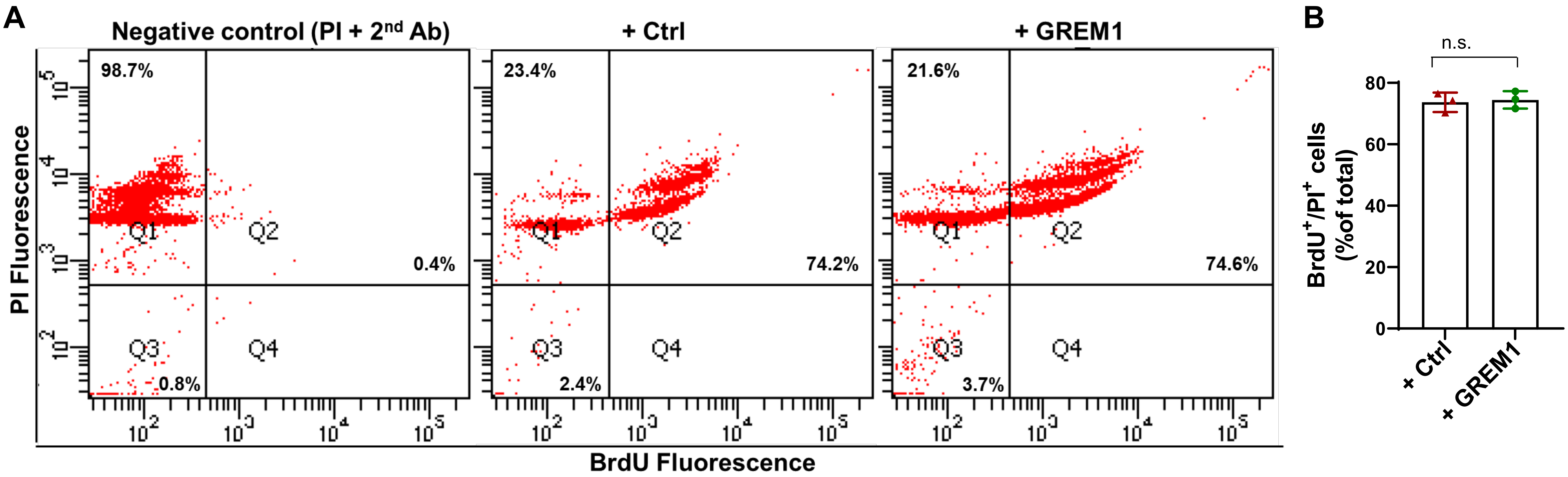

Supplemental Figure S1. Overexpression of GREM1 in hMPCs does not alter the cells' ability to proliferate. A. flow cytometry assay with BrdU and PI staining was performed to quantify the percentage of BrdU positive cells for the samples of hMPCs with or without GREM1 overexpression after 48-hour BrdU incorporation. **B**. Quantitative analysis for panel A. n.s., no significant difference.

Supplemental Figure S2. Full-length Western blot images

Figure 1B

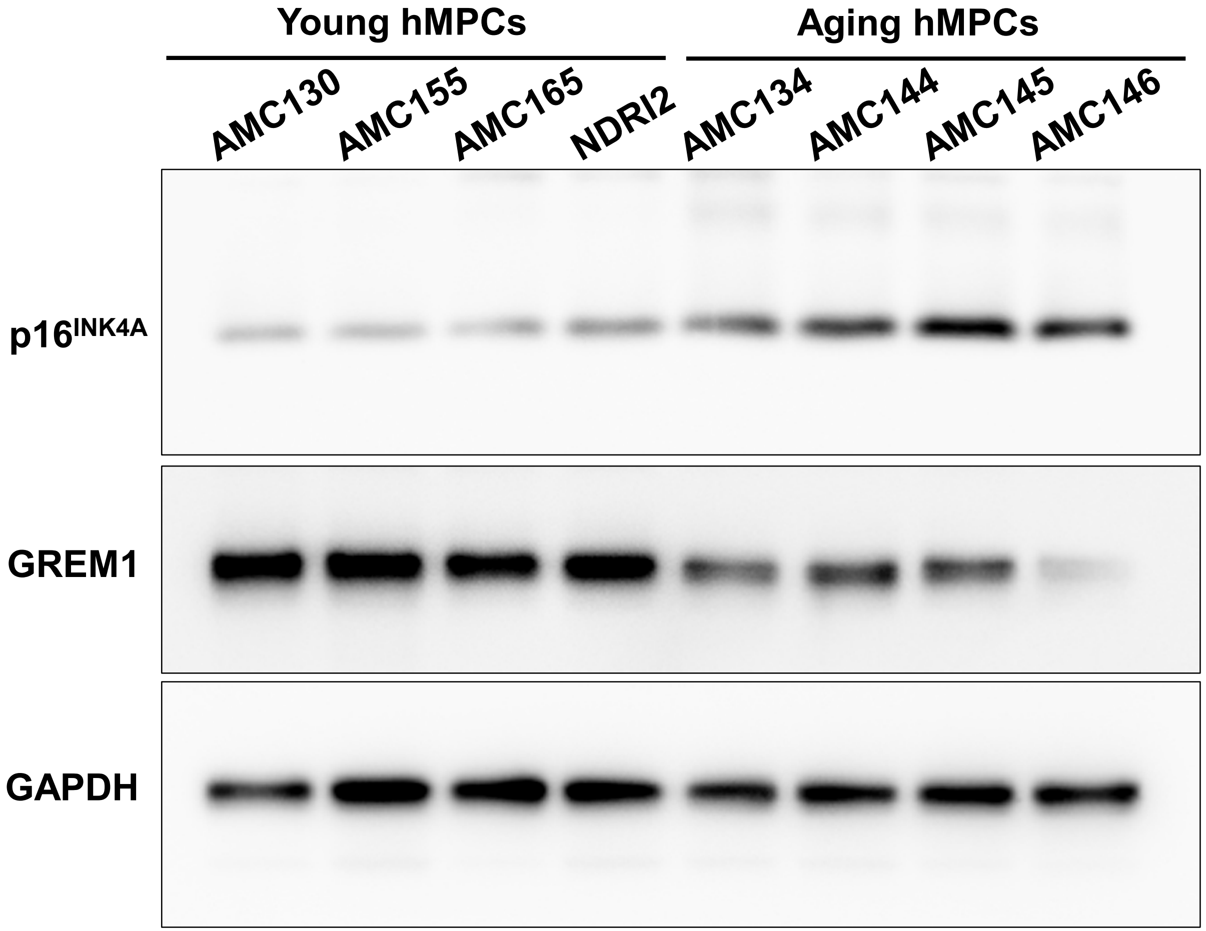

Figure 1D

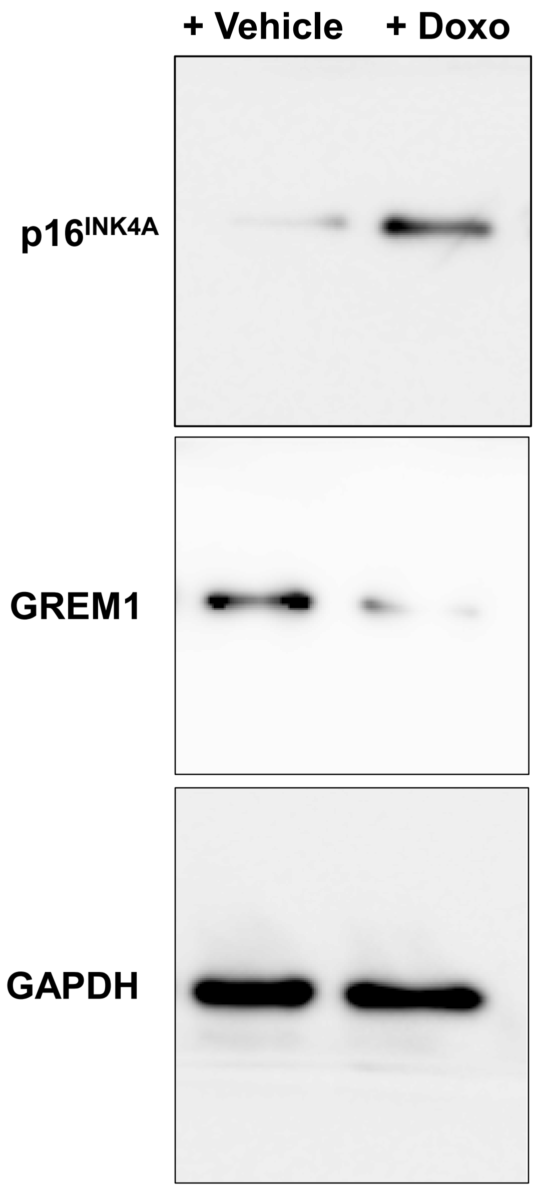

Figure 1E

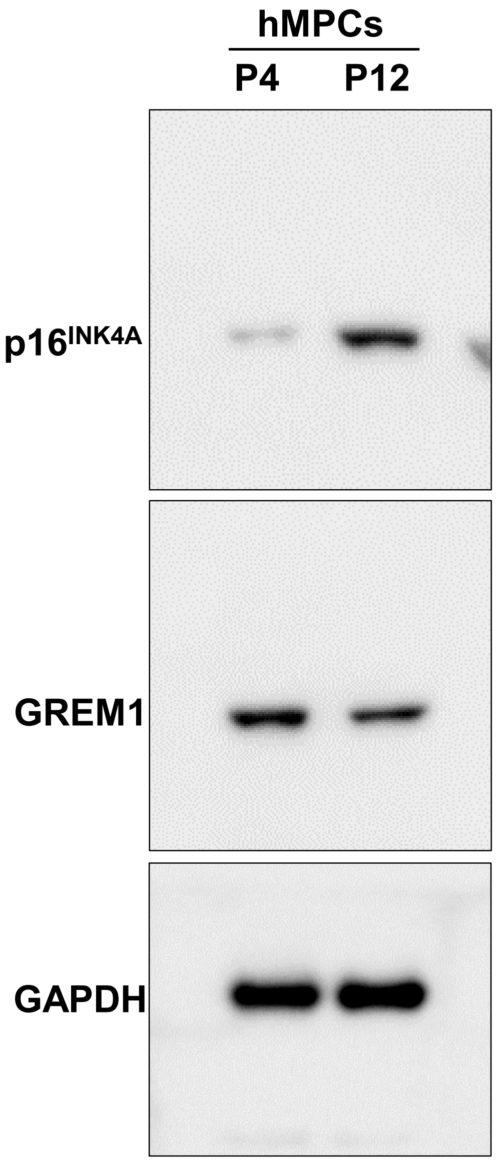

Supplemental Figure S2. full-length Western blot images (continued)

Figure 2A

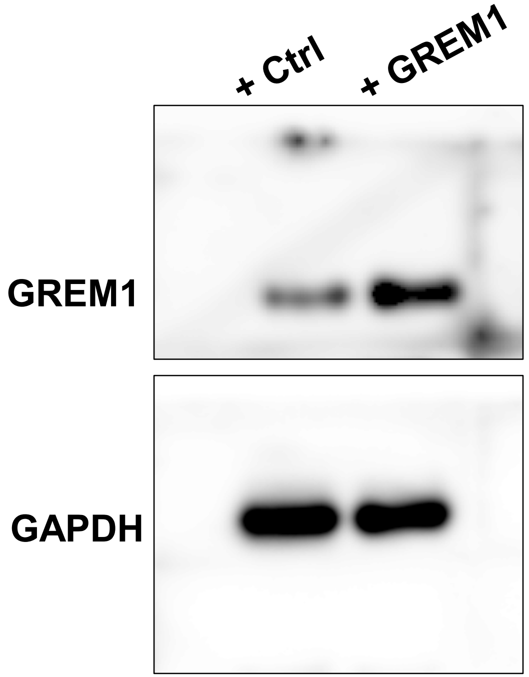

Figure 2C

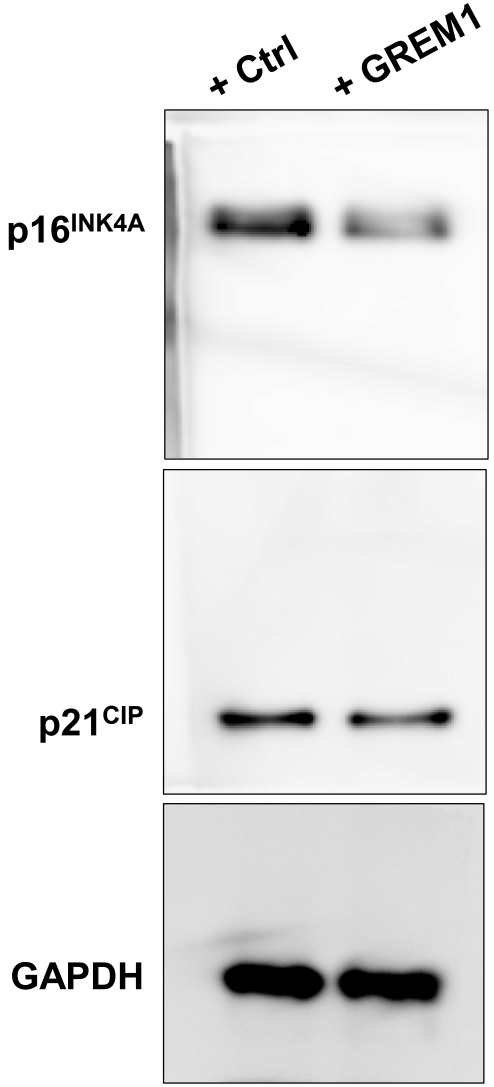

Figure 3E

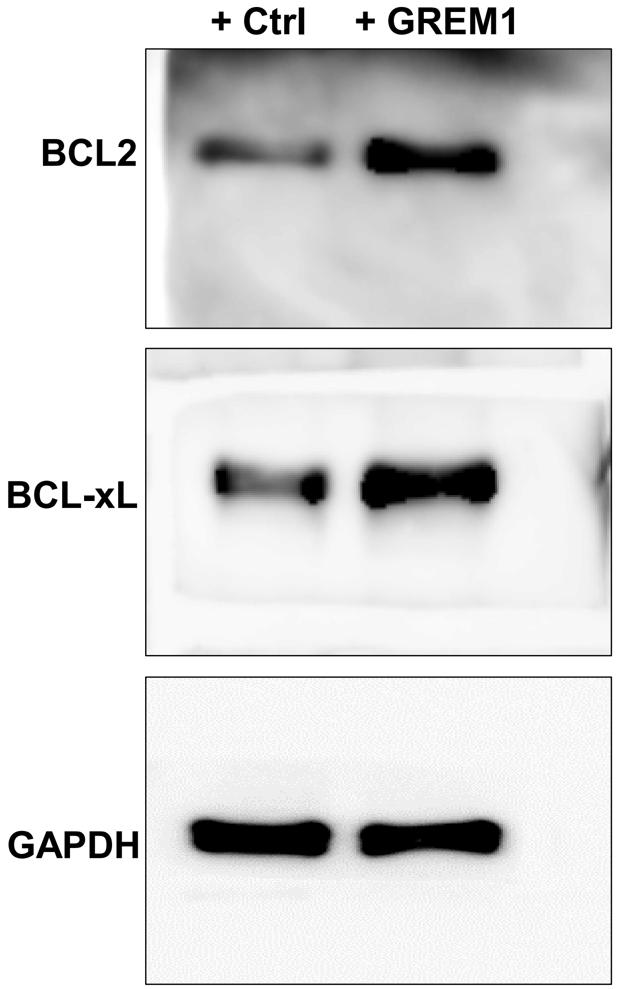

Supplemental Figure S2. full-length Western blot images (continued)

Figure 4E

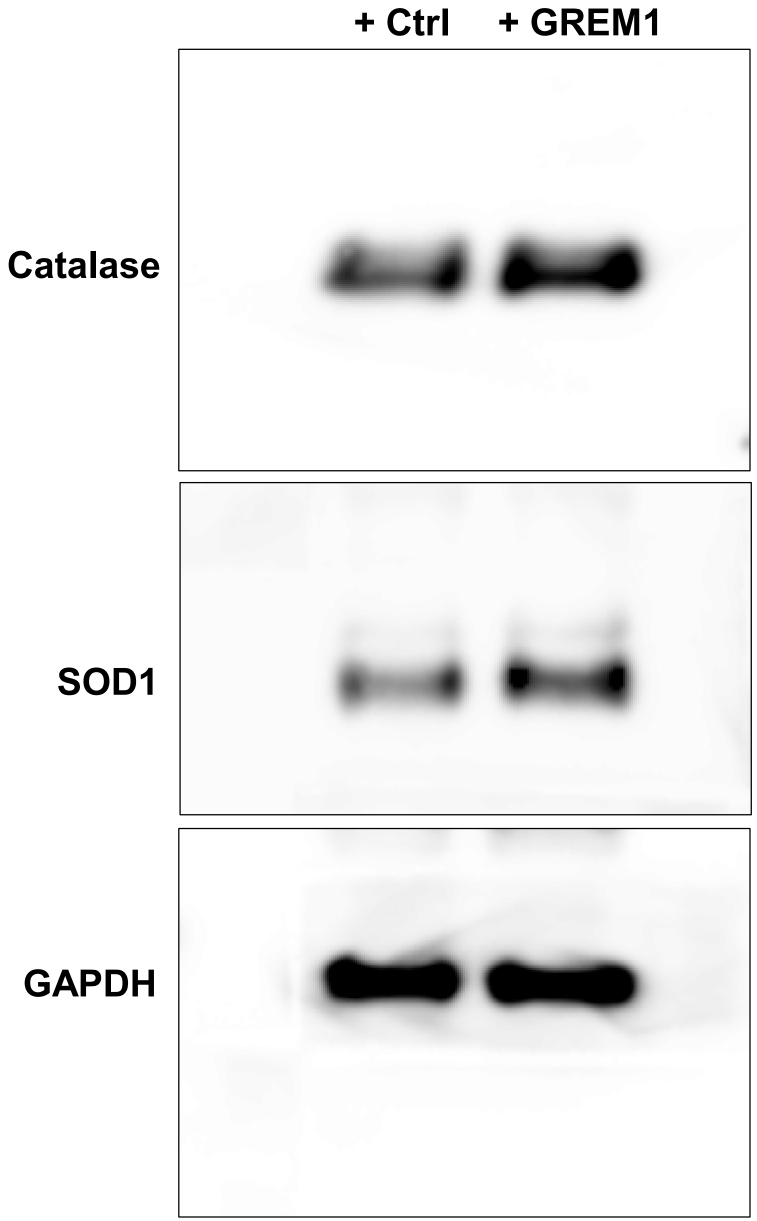

Figure 5A

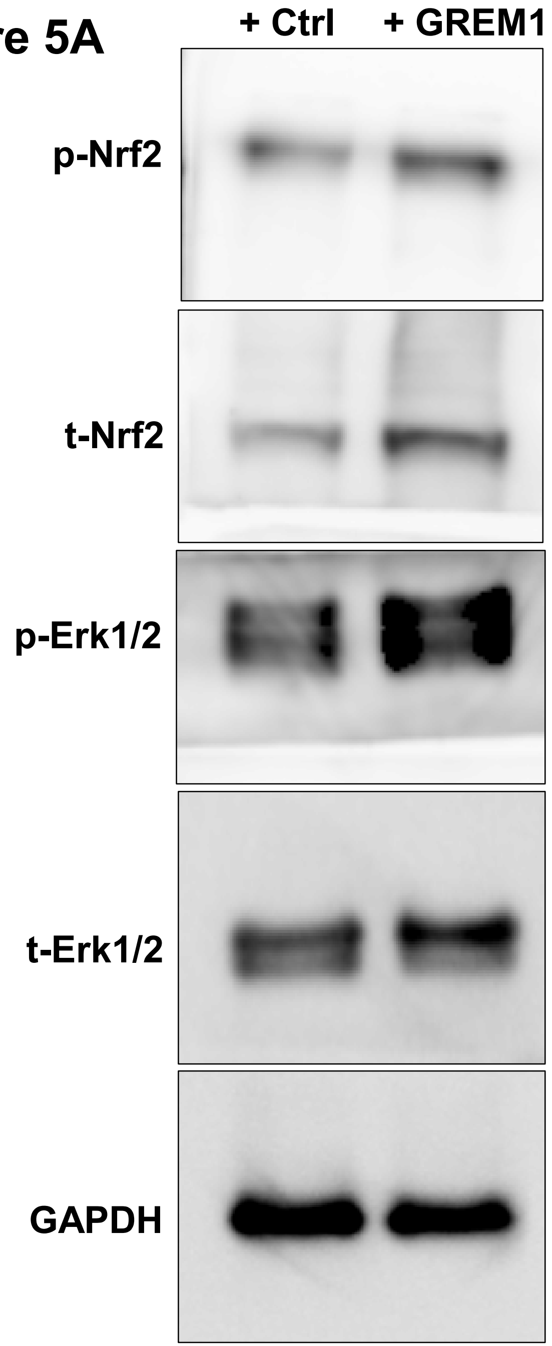

Table S1. List of primers for human genes used for the qPCR.

Genes	Forward Primer	Reverse Primer
**GREM1**	GCAGGATAGTGGAGTGAGAAAG	TCAGCCTGTGTTCTGGTATTG
**CDKN2A (p16)**	GCACATTCATGTGGGCATTT	GACTCAAGAGAAGCCAGTAACC
**CDKN1A (p21)**	CGGAACAAGGAGTCAGACATT	AGTGCCAGGAAAGACAACTAC
**FOXO1A**	TCATGTCAACCTATGGCAG	CATGGTGCTTACCGTGTG
**PSG5**	CCTGTGAACCTAAGAGTGAGAAC	TCTCGTGACACTGGGTAGAA
**BCL2L1**	TCAGGCTGCTTGGGATAAAG	AGGCTTCTGGAGGACATTTG
**BIRC3**	CAAGCCAGTTACCCTCATCTAC	CTGAATGGTCTTCTCCAGGTTC
**BCL2**	GCCAGGGTCAGAGTTAAATAGAG	GCCTCTCTTGCGGAGTATTT
**MCL1**	GTGAAGATGGTAGGGTGGAAAG	TCGGCGGGTAATCAATTCTATG
**CAT**	CTGGAGCACAGCATCCAATA	TCATTCAGCACGTTCACATAGA
**SOD1**	GGCAAAGGTGGAAATGAAGAAA	CTCAGACTACATCCAAGGGAATG
**PRDX4**	GTGGATGAGACACTACGTTTGG	CCAGCTGGATCTGGGATTATTG
**PRDX6**	CCAACCATCCCTGAAGAAGAA	GGTGTGTAGCGGAGGTATTT
**GAPDH**	GGTGTGAACCATGAGAAGTATGA	GAGTCCTTCCACGATACCAAAG

Table S2. List of antibodies against human proteins used for the Western blots.

Antigen	Catalog No.	IgG Type	Source
GREM1	ab140010	Rabbit	Abcam
$p16^{INK4A}$	92803	Rabbit	Cell signaling
$p21^{CIP}$	2947	Rabbit	Cell signaling
BCL-2	2870	Rabbit	Cell signaling
BCL2L1	2764	Rabbit	Cell signaling
SOD1	SC271014		Santa Cruz
Catalase	SC50508	Rabbit	Santa Cruz
ERK1/2	SC135900	Mouse	Santa Cruz
Phospho-ERK1/2	9154	Rabbit	Cell signaling
NRF-2	ab62352	Rabbit	Abcam
Phospho-NRF-2	ab76026	Rabbit	Abcam
GAPDH	2118	Rabbit	Cell signaling
